# Defining Advising, Coaching, and Mentoring for Student Development in Medical Education

**DOI:** 10.7759/cureus.27356

**Published:** 2022-07-27

**Authors:** Luis Santiesteban, Eric Young, Georgina C Tiarks, Maria Giulia Boemi, Raina K Patel, Kyle A Bauckman, Lauren Fine, Maria E Padilla, Vijay Rajput

**Affiliations:** 1 College of Allopathic Medicine, Nova Southeastern University Dr. Kiran C. Patel College of Allopathic Medicine, Fort Lauderdale, USA; 2 Office of Medical Education, Nova Southeastern University Dr. Kiran C. Patel College of Allopathic Medicine, Fort Lauderdale, USA

**Keywords:** mentorship program, professional mentor, undergraduate medical student, medical education, coaching, mentoring students, advising

## Abstract

Medical school curricula integrate classroom academic teaching, hands-on clinical training, longitudinal professional development, and identity formation to prepare students to enter the healthcare workforce as residents. Mentorship, coaching, and advising are well-recognized approaches used by educators to help young learners accomplish their personal and professional goals and objectives. However, undergraduate medical education literature has not clearly articulated the distinctions between the roles and core responsibilities of each guidance approach. Attempts to describe each role and responsibility have generated ambiguity and steered institutions towards implementing their own role-specific functions. The purpose of this paper is to establish a functional framework that may be used to differentiate the principal duties of a mentor, coach, and advisor in the context of undergraduate medical education (UME). Four key components are necessary to achieve this goal: (1) adopting a singular definition for each form of guidance; (2) characterizing each role based on unique skills; (3) describing the interplay between learner needs and educator capabilities; (4) training educators on how to effectively distinguish each form of guidance. Creating clear distinctions between mentors, coaches, and advisors in medical education will bolster students’ academic experience and improve the educator-learner relationship. These definitions may also benefit faculty members by providing a clear framework for their responsibilities, which can be used for evaluations or determining future promotions.

## Introduction and background

Medical education is continuously transforming with the goal of fostering innovative, competent, and ethical physicians. Modern healthcare challenges have compelled medical programs to rapidly adopt novel educational strategies to ensure that young physicians may successfully navigate complex healthcare systems [1,2].

Historically, medical training consisted of academic teaching combined with an apprenticeship under the supervision of experienced practitioners. A senior mentor would aid the young apprentice in bridging the gap between textbook theory and professional practice. As the field of medical education expands, mentors remain an integral part of student training and professional career development. Mentors impart wisdom, share expert insight, and help mentees foster skills for life-long success [3]. However, new educational cognitive strategies have permeated throughout medical education. In recent years, academic coaching and advising have become popular alternatives for individualized learning and performance enhancement [4]. Academic coaching is thought to motivate trainee introspection and increase clinical competency. A coach provides immediate corrective feedback that the student or junior doctor integrates into their practice [5]. In contrast, the function of an advisor is to assist with course scheduling, residency application, designing study schedules, and planning for research opportunities [6]. Unlike a coach, an advisor directly answers the trainee’s questions instead of promoting student self-analysis. Mentorship, coaching, and advising are widely recognized as preeminent strategies used to help medical students and new medical graduates achieve their fullest potential. Extensive work has been published on the history, benefits, and application of each guidance strategy as well as program designs for implementing each approach. One recent study established that mentor, advisor, and coach (MAC) relationships during residency can enhance resident experiences and that the majority of participants benefited from the program [7]. Meanwhile, another analysis demonstrated that guidance in medical education can increase research opportunities, strengthen professional relationships, heighten professional development, and provide insight into career decisions [8]. However, undergraduate medical literature does not provide a working framework delineating the nuances between each form of support. Consequently, these terms are mistakenly interchanged by students, educators, and administrative faculty. This problem is exacerbated when describing different parameters and attributes for mentorship, coaching, and advising in the context of medical education [9]. These parameters may include: the length and formality of the teacher-learner relationship, who benefits from the interactions, whether the trainer is an expert in the field of interest, the level of educator involvement in the student(s) evaluations, and whether feedback is involved [10,11]. This perspective encourages the conception of a common framework for defining the roles and duties of mentors, coaches, and advisors in UME by proposing the following: (1) adopting a singular definition for each form of guidance; (2) characterizing each role based on unique skills; (3) describing the interplay between common learner needs and educator capabilities; (4) training educators on how to effectively employ each form of guidance. Achieving consensus around the different characteristics of each educational approach will bolster students’ academic experience and improve the educator-learner relationship.

## Review

Defining each term

As it stands, there is no singular definition for a mentor, coach, or advisor in the context of undergraduate medical education. Although all of these terms describe a figure who guides and aids a student’s overall development, they differ based on the setting, time frame, and goals [12]. A coach, while synonymous with business and athletics, is defined as someone who encourages students to learn new skills, develop personal insight, and improve stress management [13]. Coaches may also promote self-reflection and provide feedback crucial to the development of self-assessment [12]. Meanwhile, advisors are frequently described as playing a role in helping students create study schedules, navigate specific career milestones, and plan research. Lastly, mentors are portrayed as pillars of medical education that enhance student accomplishments, job satisfaction and professional identity formation. [14]. Mentors may also provide psychosocial support, enhance well-being, and increase student satisfaction [8,15]. Mentors and advisors may utilize a more traditional, directive, senior-to-junior framework while coaching, in comparison, can be portrayed as learner-driven relationship [12].

Overgeneralized descriptions of each guidance approach generate misunderstanding amongst learners and educators. Until both the learner and faculty adopt a unified definition, the trainer-trainee relationship will suffer from mutually unfulfilled expectations [10]. Designating a formal definition for each guidance method will lay the groundwork for individualizing the terms mentor, coach, and advisor. Table 1 recommends potential definitions that can be utilized across the medical community to distinguish individual faculty roles [7].

**Table 1:** Guidance approaches and their corresponding proposed definition.

Guidance Approach	Proposed Definition
Coach	Stimulates students’ introspection and self-learning; objectively evaluates a trainee’s skillset. Supports trainees through practice and performance metrics.
Mentor	Fosters personal and professional growth by imparting wisdom, sharing experiences, and delivering expert insight. Encourages holistic long-term mentee success. Provides psychosocial support.
Advisor	Addresses questions by providing direct answers or potential solutions often based on institutional and national guidelines. Supports students with completing program-specific tasks.

Identifying unique educator characteristics

The lines between mentorship, coaching, and advising are often blurred when considering educator skillset and teaching approach. Many individuals may use these terms synonymously. However, there are vital differences between each form of guidance. Identifying their unique characteristics can significantly impact the process of building a unified framework around each approach. Table 2 demonstrates characteristics and skills for each guidance approach. While some may be specific to each role, others may overlap.

**Table 2:** Guidance approaches and their corresponding characteristics and skills.

Guidance Approach	Characteristics and Skills
Coach	Provides specific skill-oriented training
	Sessions are formal, data-driven, and focused on improving performance
	Immediate detailed feedback is given after each encounter
	Encourages self-directed learning and personal reflection
	Relationship is short-term, task dependent, and benefits mostly the student
	Always an expert in the student’s field of interest
	Not involved with trainee’s academic evaluations
Mentor	Provides holistic guidance for long-term professional and personal success
	Meetings are informal and scheduled based on mutual availability
	Combines feedback, anecdotal advice, and expert insight
	Promotes student success mostly by serving as a role model
	Usually an expert in the student’s field of interest
	Relationship is based on long-term goals, common areas of interest, mutually beneficial
	May or may not be involved in student’s academic evaluations
Advisor	Provides strategic support based on institutional or national guidelines
	Sessions are formal, task-oriented, and based on student need
	Provides direct answers or strategies to accomplish a task at hand
	Promotes strategic thinking and technical knowledge
	Frequently an expert in a specific aspect of medical education or training
	Relationship is short-term and beneficial to student
	May or may not be involved in student’s academic evaluations

Identifying learner needs

Medical education is fast paced and requires learners to acquire complex knowledge in a short time - colloquially compared to “drinking water from a fire hose.” This may generate stress. especially during the early years of medical training. It is crucial to design a concise timeline demonstrating the common challenges trainees face at different stages throughout their education path. For example, a first-year student is not as concerned with residency interviews as they are anxious about the upcoming biochemistry exam or clinical skills practicum. A timeline can serve a twofold purpose. First, it can help predict what type of support a student may need: mentorship, coaching, advising, or all three simultaneously. Secondly, a chronological map can reinforce the effort of individualizing each term on the basis of student educational needs. Intentionally pairing students and educators based on strengths and weaknesses will enrich the trainer-trainee experience by maximizing faculty involvement and student educational gain [16]. For instance, second-year medical students commonly prepare for their first national high-stakes board examination. A faculty mentor may bestow wisdom on how to cope with anxiety and the significance of maintaining focus. A mentor may help the student recognize that a board exam is just one piece of the puzzle, an item in a long checklist of requirements. The mentor may relate their story as a former student preparing for the exam and share how he/she managed to accomplish their long-term goals despite an exam score. On the other hand, a faculty coach will provide support in recognizing and targeting areas of academic weakness. If available, the coach may utilize academic metrics to track student progress and provide immediate feedback. A coach, alongside the student, will create performance goals and engage in continuous practice sessions. Lastly, a faculty advisor may assist the student with scheduling the exam, certifying that the student has met all the pre-test requirements, and confirming that the student has a full understanding of what to expect on test day. The advisor may also recommend test-related resources and information on school services like academic tutoring. Table 3 provides a general template of major challenges and experiences during a typical four-year allopathic medical school curriculum in the United States. Table 3 also postulates the type of guidance that should be prioritized given the circumstance. This chart demonstrates how each form of guidance can be employed during different events to maximize student success. Therefore, common student challenges can be a revealing element when discerning between guidance strategies.

**Table 3:** Proposed guidance approaches for common medical school challenges.

Medical School Year	Common Challenges or Tasks	Recommended Guidance Approach
1	Transition from undergraduate to medical school curriculum	Mentor, Advisor, Coach
	Adopting new study skills, increasing academic performance	Coach, Advisor
	Coping with stress, anxiety, and other psychological stressors	Mentor, Advisor
	Exploring career interests	Mentor, Advisor
	Taking on leadership roles	Mentor
	Pursuing interests outside of medicine/science	Mentor, Advisor
	Planning for research opportunities	Mentor, Advisor
	Doctoring, professional identity	Mentor
2	Board examination registration process	Advisor
	Preparing a comprehensive study plan	Coach, Advisor
	Refining studying skills and improving test performance	Coach
	Managing test related anxiety and uncertainty	Mentor
3	Selecting clinical rotations	Mentor, Advisor
	Preparing for Shelf Exams	Coach, Advisor
	Developing a specific set of clinical skills	Coach, Mentor
	Process of obtaining letters of recommendation	Advisor, Mentor
	Exploring specialty of interest	Mentor, Advisor
4	Choosing Elective Rotations	Mentor, Advisor
	Refining a specific set of clinical skills	Coach
	Planning fourth-year research	Mentor, Advisor
	Preparing for board examination	Coach
	Residency application process	Advisor
	Preparation for residency interview	Mentor, Advisor

Training educators

The constantly changing medical education landscape has rendered any single form of guidance inadequate in satisfying the learner’s professional and personal development. Medical students and young physicians depend on experienced faculty who can seamlessly maneuver between each form of guidance. To that end, faculty development programs play a critical role in creating a conventional framework for distinguishing between each guidance approach. Training educators on how to target student deficits by utilizing the most effective guidance strategy will help decrease role confusion. Faculty development programs may also contribute towards standardizing the way faculty learn about mentorship, coaching, and advising in medical education. Lastly, faculty training programs can help young educators navigate the dynamic teacher-learner relationship by pairing them with the appropriate faculty. Essentially, this partnership will generate a robust teacher-teacher and teacher-learner network that will enhance the learning process [17]. Overall, faculty development can help create a community-wide consensus on what each educational approach consists of and how they need to be separated by skills and capacity to help students.

## Conclusions

Medical education is a dynamic process that encompasses formal academic training as well as guidance from experienced faculty members. Historically, mentors assisted young students and new medical graduates in their personal and professional journeys. Mentors continue to serve as role models who help students find and develop their professional identities. In recent decades, medical programs have approved academic coaching and advising as a way of improving student learning. Although each medical school has a distinctive vision, mission, and set of values, a unified understanding of the key roles and responsibilities would benefit both students and educators. Creating a comprehensive framework for each approach will necessitate the creation of common role definitions, appreciation for each role’s unique characteristics and skills, knowledge of the interplay between students’ needs and educators’ abilities, and faculty development programs that will train educators to recognize the differences of each form of guidance.
